# The Learning to Be Project: An Intervention for Spanish Students in Primary Education

**DOI:** 10.3389/fpsyg.2021.632617

**Published:** 2021-04-30

**Authors:** Davinia M. Resurrección, Óliver Jiménez, Esther Menor, Desireé Ruiz-Aranda

**Affiliations:** ^1^Department of Psychology, Universidad Loyola Andalucía, Dos Hermanas, Spain; ^2^Department of Personality, Assessment and Psychological Treatment, Universidad de Málaga, Málaga, Spain; ^3^Department of Communication and Education, Universidad Loyola Andalucía, Dos Hermanas, Spain

**Keywords:** social emotional learning, socioemotional competencies, learning to be, longitudinal study, educational policies

## Abstract

Despite the emphasis placed by most curricula in the development of social and emotional competencies in education, there seems to be a general lack of knowledge of methods that integrate strategies for assessing these competencies into existing educational practices. Previous research has shown that the development of social and emotional competencies in children has multiple benefits, as they seem to contribute to better physical and mental health, an increase in academic motivation, and the well-being and healthy social progress of children. This study aims at assessing the possible changes in children’s self-esteem, socio-emotional competencies, and school-related variables after participating in the Learning to Be project (L2B) project.

**Methods:** This quasi-experimental study included an intervention group (L2B) and a control group. The participants were 221 students in primary education (55.2% girls) between the ages of eight and 11 (*M* = 9.31; SD = 0.89). The L2B intervention program took place over a period of 5 months. The assessment was carried out twice, before and after the intervention through three main evaluation instruments: the Rosenberg’s Self-Esteem questionnaire, the Socio-Emotional competence questionnaire (SEQ), and self-report scales for measuring school difficulties, school engagement, opinions about school, and school absence. Ten schools from different Spanish provinces participated.

**Results:** The results indicate that those participants in the experimental group show higher self-esteem, better responsible decisions, and higher self-awareness than those in the control group. There were no other statistical differences between groups.

**Conclusions:** The results of this work suggest that the implementation of the L2B program did not improve social and emotional competencies in primary school students. Further research related to how include formative assessment in SEL programs is needed.

## Introduction

The term Social and Emotional Learning (SEL), which describes a framework that encompasses the acquisition of personal and positive relationship skills (CASEL, 2013; Schonert-Reichl, 2019), was first introduced in 1994. In this sense, school based SEL involves the implementation of policy practices to promote social, personal, and ethical behaviors in both students and teachers (Taylor et al., 2017). Following The Collaborative for Academic, Social and Emotional Learning (CASEL) guide, SEL interventions are focused on five interrelated competencies clustered into self-awareness, self-management, social awareness, relationship skills, and responsible decision making (CASEL, 2015; Weissberg et al., 2015; Abrahams et al., 2019). SEL includes, among others, the process of developing effective communication skills, of cooperating with peers, and of solving problems, recognizing and regulating emotions effectively (CASEL, 2005). Although in a variety of formats, most SEL programs are delivered by teachers during school hours (Wigelsworth et al., 2016).

Among the competencies suggested by CASEL for SEL interventions, the principles of self-awareness and self-management are related to students’ approaches toward the self (self-esteem, confidence, persistence, self-efficacy, and self-concept) and to achievable personal and academic goals. These competencies have proven to be protective factors as they reduce the probability of problem behaviors (Catalano et al., 2003) and increase the probability of success at school and later in life (Clarke et al., 2015; Weissberg et al., 2015). Regarding the effects of CASEL programs, recent systematic reviews and meta-analyses have shown the major benefits of SEL programs at schools (Corcoran et al., 2018; Mahoney et al., 2018). Several meta-analyses highlight that SEL interventions improve prosocial behavior, promoting academic performance as well as enhancing positive self-esteem. The same studies reveal that these interventions serve as a protective factor against several problems, such as emotional distress, mental problems, or drug use (Durlak et al., 2011; Sklad et al., 2012; Wigelsworth et al., 2016; Taylor et al., 2017). In addition, the meta-analysis carried out by Taylor et al. (2017) finds that the achieved improvements reported in the included studies remain after several years. Finally, the results reported by these meta-analyses highlight that the main outcomes for SEL programs are clustered into social behaviors, emotional problems, and academic achievement (Weare and Nind, 2011).

Despite the positive results obtained with the implementation of SEL in several schools across different countries, there is a lack of a general model that would integrate SEL into the educational curricula. On the contrary, and as a result, a wide diversity of approaches to social and emotional competencies are being developed (Cefai et al., 2018). The European Union Analytical Report on social and emotional education highlights that the vast majority of research and socio-emotional interventions have taken place in the United States. Specifically, in Spain, the legal educational framework integrates emotional competencies into the curriculum, although it does not stipulate their implementation and development (BOE, 2006). It would be, thus, necessary to develop a training program in SEL for the whole educational community, following the recommendations given by the European Union (Cefai et al., 2018). Furthermore, previous studies have pointed out cultural differences in the way of expressing, interpreting, and giving meaning to emotions (Hoemann et al., 2020) as these emotions should not be considered mere individual experiences, but experiences constructed in social interactions and influenced by cultural contexts (Holodynski and Friedlmeier, 2006). Therefore, blind adoption of United States models without appropriate cultural adaptation could be problematic.

Taking this into account, the project Learning to Be (L2B) adapted SEL standards from Illinois State Board of Education (Illinois State Board of Education (ISBE), 2018) and proposed a framework for SEL implementation at schools in Europe [for a review of the project see Aguilar et al. (2019)]. This project was an opportunity to contribute to the development of innovative educational policies at both national and international levels.

### The Learning to Be Project

Despite the importance of social and emotional competencies in education, acknowledged by most national curricula, how to assess those competencies and how to integrate assessment strategies into education practices were yet to be determined. There are several practical approaches to work with socio-emotional competences such as the INTEMO or “Aprende a convivir” program, among others (Ruiz-Aranda et al., 2008; Alba et al., 2015). However, it is also necessary a common understanding of what social and emotional competencies were and how they could be encouraged and assessed. Social and emotional competencies must be understood as the set of knowledge, capacities, abilities, and attitudes necessary to understand, express, and regulate social and emotional phenomena appropriately (Bisquerra and Pérez, 2007).

For these reasons, the main objective of the L2B project was to design a formative assessment method and the tools necessary for the development and evaluation of social and emotional competencies at primary and secondary schools. L2B project aims at overcoming limitations of previous programs in terms of evaluation. In order to achieve this goal, seven European countries developed the “Toolkit for Assessing Social and Emotional Skills at School” (Agliati et al., 2020) which could serve as a theoretical and methodological model for assessing social, emotional, and health-related skills. This Toolkit is designed for teachers in primary and secondary schools without distinction of the subjects where it would be applied. In addition, it can also be used by other educators in non-formal contexts. The Toolkit includes the theoretical introduction related to what socio-emotional competences are; description of teaching methods, assessment tools for teachers and students, and socio-emotional learning standards for two different age groups, (i.e., primary and secondary school students).

The SEL model developed by the L2B project presents some key elements: Social and Emotional Competencies (SEC), Social and Emotional Learning (SEL) Standards; Social and Emotional Learning in Class and Assessment Practices. The toolkit provides teachers, managers and students with self-assessment tools so that they can evaluate the SEL situation of the school. In addition, it offers tools that educators can use to track progress and to monitor the achievement of social and emotional competencies in their students, to foster learning experiences compatible with these competencies and to check on their students’ learning development. The whole project is based on a formative assessment approach, that is to say, a constant process of assessment and feedback in class that helps teachers and students track their progress and identify areas and needs for improvement. It is an active practice that can be applied for assessing not only subject-based learning but also the development of social and emotional skills in the classroom. This Toolkit presents an easy-to-follow model of Formative Assessment developed by Dylan Wiliam (2011) and expressed in five main strategies to be used in class: (1) clarifying, sharing, and understanding learning intentions and criteria for success (2) eliciting evidence of student learning (3) providing feedback that moves student forward (4) activating learners as instructional resources for one another (5) activating learners as the owners of their learning.

The L2B project was carried out in five countries (Italy, Latvia, Lithuania, Slovenia, and Spain). Each country selected twenty schools (ten primary schools and ten secondary schools) to be into the control group or the experimental group. The main goal of the project was to develop and implement the Toolkit in the experimental schools, so the teaching staff and management teams of those schools received specific training in the development of socio-emotional competencies. In tandem with this effort, the project intended to develop a SEL model which would impact the European educational policies.

### The Current Study

Our study aims to assess if there are changes in socio-emotional competencies, self-esteem and school-related variables after participating in the L2B project. It was hypothesized that those participants in the experimental group would show higher socio-emotional competencies and self-esteem than those participants in the control group.

## Materials and Methods

### Participants

A convenience sample of ten primary education schools located in different Spanish provinces participated in the study, half of which were state schools and the other half private. School principals of these schools were contacted and asked to join the project. Following a detailed explanation of goals and intended activities, the schools decided whether they wanted to be part of the experimental or of the control group. As such, it is a non-randomized quasi-experimental study. The main criterion for the selection of school centers was their non-involvement in any other SEL programs. After signing the agreement with the school, students’ parents were informed of the project and asked for written consent for the participation of children. The participants were 221 primary education students, (55.2% girls) aged between 8 and 11 years old (*M* = 9.31; SD = 0.89). Schools were divided into experimental (*n* = 103) and control schools (*n* = 118). The median age was 8.72 years (SD = 0.67) for the control schools and 9.82 (SD = 0.72) for the experimental schools.

### Instruments

The following instruments were employed:

- Social Emotional Competence Questionnaire (SECQ; Zhou and Ee, 2012): this 25-item with a 7-point Likert-type response option (1 = not at all true of me to 7 = very true of me) instrument includes the five dimensions based on SEL model (i.e., self-awareness, social awareness, self-management, relationship management, and responsible decision-making). Example items of each scale were: (a) I know what I am thinking and doing; (b) I understand why people react the way they do; (c) I can stay calm in stressful situations; (d) I am tolerant of my friend’s mistakes; (e) I weigh the strengths of the situation before deciding what I will do. The scale showed good reliability in each subscale (Cronbach’s α: self-awareness: 0.64, social awareness: 0.72, self-management: 0.73, relationship management: 0.69, responsible decision-making: 0.76, and total scale: 0.86).

- Self-esteem Scale (Rosenberg, 1979; Tuominen-Soini et al., 2008): the short version of the scale includes five items reflecting self-acceptance, self-respect, and overall attitude toward oneself with a 7-point Likert-type response option (1 = totally disagree to 7 = totally agree). The scale showed medium reliability (α = 0.57). The items were: (a) I feel I have a number of good qualities; (b) Sometimes I think I am no good at all; (c) I take a positive attitude toward myself; (d) I wish I could respect myself more; (e) All in all I am satisfied with myself.

Socio-demographic information, such as age (as an open-ended question) and gender (as boy, girl, or I do not want to tell), was assessed. In addition, variables related to the school context were self-reported by the students. The questions included were taken from Finnish School Health Promotion Study (2017). The original version of the questions went through a back-translation process from the original language to Spanish and from Spanish to the original language. Both versions were then compared. Due to the low alphas in the sum scores based on the school domain items, items as separate have been used. The questions were as follows:

- School difficulties: participants were asked about difficulties when doing tasks related to reading and writing. Responses ranged from 1 (Not at all) to 5 (Very much). The items were: Do you have difficulties (a) following the classes?; (b) doing tasks that require writing?; (c) doing tasks that require reading?

- School engagement: participants were asked for their opinion on going to school and their feelings concerning schoolwork. Responses ranged from 1 (completely disagree) to 5 (completely agree). The items were: (a) I like being at school; (b) I am often tired; (c) I am often excited about schoolwork; (d) There is no point in going to school; (e) I cannot cope at school.

- Opinions about school: participants were asked for their opinion on studying and about their school. Responses ranged from 1 (completely disagree) to 5 (completely agree). The items were: (a) I feel happy in my school; (b) I feel comfortable in my school; (c) Teachers encourage me to express my opinion in class; (d) Teachers are interested in how I’m feeling; (e) The teachers treat us pupils fairly; (f) I can have an influence on issues and decisions in my school; (g) I feel my teachers accept me as I am; (h) I feel that most of my teachers care about me; (i) I feel a lot of trust in most of my teachers.

- School absence: this scale assessed how many days in the previous month the participants had been absent from school and the different reasons why they had missed school: (a) due to illness; (b) due to skipping class on purpose; (c) due to other reasons. Responses ranged from 1 (none) to 4 (more than 5 days).

### Procedure

Ethical Boards of both the University of Helsinki and the Universidad Loyola Andalucía approved the study. Schools participated in the research by completing survey questionnaires which were held twice, once in September 2018 and a second time in May 2019 (i.e., at the end of the school year). Both control and experimental groups completed the questionnaires before the teachers training, so that the study avoided any influences derived from the participation in the training. Questionnaires were answered at the computer rooms of the schools. The number of students answering the questionnaires at the same time (ranging from 5 to 25) varied according to the capacity of the room. The assessment lasted 20 min approximately. All the students responded to the questionnaire using the online platform Survey Gizmo, while they were supervised by their teacher tutor and a member of the project team.

### L2B Experimental Group

The Toolkit includes a theoretical component on SEL, a description of teaching methods that follow a formative assessment approach, assessment tools for both teachers and students, and SEL standards. Specifically, the Toolkit includes descriptions of different instructional teaching strategies such as setting learning goals, and practical SEL assessment tools at individual, group and whole-school levels.

Once the different school groups (students, teaching and non-teaching staff) had been assessed, teachers, psychologists, social workers and teacher assistants of each school in the experimental group were trained for 16 h on SEL principles, on formative assessment and on the techniques described in the manual. This training, which took place after the initial assessment, was delivered by psychologist and teachers with previous experience in SEL competencies who in turn had received specific training on the Toolkit (see Table 1). After the training, and while SEL was being implemented in the school, the teaching staff received regular support. Centers were periodically visited by supervisors who offered guidance on the use of methods and tools for socio-emotional skills learning and assessment. The teachers implemented the model described in the Toolkit in different sessions throughout 5 months. As the program follows a formative assessment initiative, the teacher could work on the different concepts addressed in the Toolkit as they were demanded by specific situations in class. In this continuous process, the teachers collected and analyzed different learning evidence and adjusted their teaching accordingly, so that learning would improve, and student progress encouraged. In other words, the teacher identified and set targets for growth in every lesson (Agliati et al., 2020).

**TABLE 1:** Contents of each training session.

**Session (hours)**	**Contents**
One (5 h)	1. Participants and L2B project presentation2. Definition of SEL 3. SEL competencies and benefits of SEL4. Toolkit presentation and introduction to the manual
Two (5 h)	1. SEL standards according to each group of age2. Working with guidelines for assessing students (content included in the Toolkit)3. Learning to work with group presentation rubrics (content included in the Toolkit)4. Formative assessment practices for classroom use
Three (6 h)	1. Teaching methods to strengthen SEL and formative assessment strategies2. Classroom strategies to maximize the teaching and reinforcement of SEL competencies3. Practical application to each class by their teacher

### Control Group

Schools in the control group were assigned to a waiting list and received the intervention training after a 5-month waiting period.

### Design

A quasi-experimental design, with control-group and pre-test/post-test, was used. A quasi-experimental design was used because the schools were not randomly assigned to the groups. The research was conducted over a period of 10 months (from September 2018 to May 2019). It first started with the assessment (pre-test) of both teachers and students, followed by the training offered to teachers in the experimental group. The 5-month intervention program took then place and, finally, schools participated in the post-intervention assessment.

### Data Analysis

The data were analyzed as follows: in order to reduce the bias caused by the differences between groups (control and experimental), the analysis followed a multi-group design with covariates (ANCOVA). This design allows to control the effect of pre-test scores (covariates) in the relationship between the dependent and the independent variables, minimizing the variation due to the ANOVA error term (Tabachnick and Fidell, 2007). Possible gender and age differences were analyzed in order to know the existence of other possible covariates. Independent *t*-test were carried to analyze differences in self-reported school domain. Afterward, we checked the assumptions of normality, variance homogeneity, and error-free measurement of covariates, the independence between the indirect variable and the covariate, as well as the linearity between the depending variables. When assumptions were not fulfilled, non-parametric Mann–Whitney *U* was carried out.

## Results

Since all the measures were self-reported, common method bias was established using Harman’s single factor test. We found a single factor contributing less than 50% in variance (χ^2^ = 17.378) (Podsakoff et al., 2003).

In order to determine whether there were differences in the post-intervention due to the age and gender of participants, data were analyzed by analysis of covariance, using the pre-test results as a covariate. The assumptions of normality, variance homogeneity and the homogeneity of regression coefficients were met. Regarding gender, the analysis showed that there was no statistically significant interaction between gender and self-esteem [*F*(2, 217) = 0.92, *p* = 0.39, η*^2^* partial = 0.00] or between gender and total SEC [*F*(2, 215) = 1.92, *p* = 0.14, η*^2^* partial = 0.01]. Regarding age, the analysis showed that there was no statistically significant interaction between age and self-esteem, whilst controlling for pre-test self-esteem scores [*F*(3, 216) = 2.41, *p* = 0.06, η*^2^* partial = 0.03]. However, there were a significant effect of age on the SEC [*F*(3, 214) = 4.96, *p* = 0.00, η*^2^* partial = 0.06] after controlling pre-intervention SEC scores. Participants with 11 years old showed less total SEC (*M* = 102.05, SD = 17.14) when comparing with younger participants [10 years old (*M* = 119.30, SD = 15.48), 9 years old (*M* = 118.74, SD = 15.22), and 8 years old (*M* = 117.68, SD = 17.40)]. When analyzing SEC subscales, there was statistically significant differences in Self-Awareness [*F*(3, 216) = 4.59, *p* < *0.00*, η^2^ partial = 0.06], showing less total score among 11 years old participants (*M* = 23.25, SD = 5.01) when comparing with younger participants [10 years old (*M* = 26.43, SD = 4.08), 9 years old (*M* = 26.71, SD = 3.30), and 8 years old (*M* = 26.90, SD = 2.87)]. In addition, there was statistically significant differences in Self-Management subscale [*F*(3, 215) = 6.41, *p* < 0.001, η*^2^* partial = 0.08] showing less total score among 11 years old participants (*M* = 17.36, SD = 7.19) when comparing with younger participants [10 years old (*M* = 23.80, SD = 4.64), and 9 years old (*M* = 22.20, SD = 5.58)]. There were no statistically significant differences in the rest of subscales; Social Awareness [*F*(3, 216) = 1.02, *p* = 0.38, η^2^ partial = 0.01], Relationship Management [*F*(3, 215) = 1.90, *p* = 0.13, η^2^ partial = 0.02] and Responsible decision-making [*F*(3, 214) = 2.07, *p* = 0.10, η^2^ partial = 0.02].

In order to assess whether there were differences between the control group and the experimental group in self-reported school variables, independent *t*-test analyses were carried out (see Table 2). Results showed that there was no statistically significant differences for School Engagement [*t*(213) = 0.85; *p* = 0.19], Opinions about school [*t*(211) = 1.42; *p* = 0.07], and School absence [*t*(203) = 0.44; *p* = 0.32]. However, participants at the experimental group (*M* = 6.17, SD = 4.27) compared to the control group (*M* = 4.93; SD = 3.08) showed greater School difficulties [*t*(172.34) = 2.40; *p* = 0.00 *d* = 0.33].

**TABLE 2:** *T*-test descriptive statistics.

**Variables**	**Experimental group**		**Control group**	
	**Pre-test**	**Post-test**	**Pre-test**	**Post-test**
**School engagement**				
I like being at school	4.36 (0.93)	4.34 (0.92)	4.18 (0.89)	4.04 (0.93)
I am often tired	3.25 (1.23)	2.99 (1.22)	3.20 (1.22)	3.21 (1.21)
I am often excited about schoolwork	3.50 (1.17)	3.36 (1.16)	3.25 (1.22)	3.19 (1.13)
There is no point in going to school	4.49 (1.05)	4.69 (0.90)	4.50 (1.02)	4.66 (0.70)
I cannot cope at school	3.92 (1.42)	4.19 (1.34)	4.00 (1.19)	4.02 (1.14)
**Opinions about school**				
I feel happy in my school	4.76 (0.70)	4.76 (0.55)	4.57 (0.83)	4.56 (0.73)
I feel comfortable in my school.	4.70 (0.72)	4.70 (0.73)	4.59 (0.74)	4.55 (0.82)
Teachers encourage me to express my opinion in class.	4.42 (0.87)	4.43 (0.98)	4.40 (0.76)	4.31 (0.92)
Teachers are interested in how I’m feeling	4.41 (0.90)	4.40 (0.97)	4.42 (0.83)	4.37 (0.85)
The teachers treat us pupils fairly.	4.40 (1.02)	4.29 (1.05)	4.43 (0.94)	4.37 (0.93)
I can have an influence on issues and decisions in my school.	3.74 (1.30)	4.12 (1.05)	3.75 (1.06)	3.56 (1.21)
I feel my teachers accept me as I am.	4.75 (0.65)	4.69 (0.82)	4.65 (0.77)	4.61 (0.75)
I feel that most of my teachers care about me.	4.39 (0.92)	40.46 (0.97)	4.39 (0.93)	4.43 (0.86)
I feel a lot of trust in most of my teachers	4.66 (0.75)	4.61 (0.85)	4.56 (0.86)	4.48 (0.80)
**School absence**				
Due to illness	1.51 (0.72)	1.56 (0.75)	1.36 (0.65)	1.62 (0.78)
Due to skipping the class by purpose	1.01 (0.10)	1.03 (0.17)	1.05 (0.28)	1.05 (0.26)
Due to other reason	1.18 (0.50)	1.45 (0.72)	1.19 (0.51)	1.30 (0.59)
**School difficulties**				
Following teaching in class?	1.89 (1.47)	1.93 (1.53)	1.71 (1.32)	1.74 (1.30)
Doing tasks that require writing?	1.99 (1.44)	2.17 (1.56)	1.69 (1.25)	1.72 (1.16)
Doing tasks that require reading?	1.84 (1.46)	2.06 (1.66)	1.83 (1.45)	1.47 (1.14)

*p < 0.05.

To assess differences between pre-post scores, a between-groups covariance analysis (ANCOVA) was carried out. The intervention was considered as the independent variable (control group and experimental group), and the results corresponding to the dependent variables of the pre-test were considered covariables. Thus, the differences between groups were estimated with the differences in pre-test results removed. ANCOVA analysis showed no statistically significant differences at the total SEC scores. Non-parametric Mann–Whitney *U* test was carried out for Self-Awareness subscale. Self-awareness at the experimental group scores (MR = 127.77) were higher than those at the control group (MR = 96.36), showing that this difference was statistically significant (*U* = 4349.5, *p* < *0.001*) (see Tables 3, 4). In absence of reactivity, experimental group showed higher self-esteem and higher Responsible decision-making subscale scores when comparing with the control group after the intervention.

**TABLE 3:** ANCOVA results variables.

	***F***	***p***	**η*^2^ partial***
Self-esteem	4.39	0.03*	0.03
SEC	2.15	0.14	0.01
Social awareness	0.23	0.63	0.00
Self-management	0.14	0.70	0.00
Relationship management	0.64	0.42	0.00
Responsible decision-making	5.17	0.02*	0.02

SEC, Social Emotional Competence; *p < 0.05.

**TABLE 4:** Descriptive statistics for each group.

**Variables**	**Control group**			**Experimental group**		
	***M***	***SD***	***M* adjusted**	***M***	***SD***	***M* adjusted**
Self-esteem	24.83	4.66	24.86	26.25	4.75	26.12
SEC	114.44	15.94	115.89	120.60	16.52	124.75
Social awareness	21.33	5.08	21.69	22.46	6.20	22.06
Self-management	21.82	5.58	22.21	22.94	6.10	22.50
Relationship management	25.81	3.94	25.89	26.37	3.76	26.29
Responsible decision-making	20.19	3.3	20.36	21.48	2.84	21.30

*SEC, Social Emotional Competence.*

## Discussion

The aim of this study was to analyze possible changes in self-esteem, socio-emotional competencies and school-related outcomes of primary school students after their participation in the L2B project. Following the results, we have found that the initial hypotheses have only been fulfilled partially, as those students who participated in the intervention improved in relation to self-esteem, self-awareness, and responsible decision-making, while the results in school-related domain and in total score of socio-emotional competencies were not in line with our initial expectations. These results are consistent with those presented by previous studies (Brackett et al., 2012) and suggest that students with greater emotional and social competences may use the information provided by emotions to guide attention into appropriate thoughts and make better decisions and improve their psychological functioning. A possible explanation for these results might be related to age differences in the benefits of participating in the L2B program. Some studies have suggested differences in the emotional maturation of some competencies, such as the expression or emotional regulation, showing differences from 9 to 10 years (Jones et al., 1998; Gordillo et al., 2015). Thus, there might exist a change in diverse social and emotional competences, such as self-awareness and self-management.

One of the main results pointed that there were no differences in the overall score of social and emotional competencies after participating in the L2B program. This result might be explained due to the employment of a formative assessment approach. Following this framework, teachers at the intervention group needed to recognize students’ development to implement their socio-emotional learning in their classrooms. Despite the training, some teachers may have felt they did not possess enough knowledge to improve and to assess their students’ improvement in SEL competencies (Corcoran et al., 2018).

The increase in the items reporting school difficulties in the experimental group can also be due to the students’ awareness of their behavior, an indication that further evaluation tools which would allow a more global vision of the situation are required. A 360-degree evaluation (including parents, teachers, and students) could be useful for the implementation of this kind of programs (Bisquerra et al., 2006; Silva and Martorell, 2018). It would also be useful to know if these results would remain the same in the long term, or, on the contrary, they would improve as they were a consequence of the implementation of SEL in the ordinary teaching practice. Regarding to school opinions and school absence, we did not find differences between the groups after the intervention. The lack of differences may be because these concepts were not directly worked on with the Toolkit, even when it was an expected result if socio-emotional competencies were to improve (Brackett et al., 2004). Although the intervention is based on a manual, as suggested by Weare and Nind (2011), the Toolkit allows for an open implementation in accordance with the necessities observed by the teacher. This may have resulted in differences in the applied contents and the degree of implementation. Thus, despite main principles for social and emotional learning and practical techniques were provided, there was no specific instructions in the experimental group to follow the same activities, creating possible differences among teachers in their application in their classes. Additionally, the application of the Toolkit may have been influenced by characteristics of the schools such as the number of students in a room or the skill of the teachers, who could need a longer period of training, as is already the case for other related studies (Body et al., 2016; Mira-Galvañ and Gilar-Corbi, 2020). For this reason, it would be necessary to evaluate in depth the use of the Toolkit in further groups of teachers after they receive longer training (i.e., more than 20 h), knowing in advance whether the teachers have efficiently acquired the skills for a subsequent application in the room. In this regard, it would be advisable to assess when the Toolkit could be best implemented, since its application in critical moments of the academic course could influence in the final results, as it is mentioned in the Research Progress Report of the Learning to Be Project (Berg et al., 2020). In view of the above, it would be essential the teacher participation in the planning, implementation and evaluation of the SEL program in future studies (Zins et al., 2004). The L2B project was developed to improve and assess social behavior, emotional problems, and academic achievement. In this line, the present study has sought to verify if the implementation of the Toolkit developed in the Learning to Be project for students in primary education is effective in the improvement of the skills mentioned above.

In addition to the flaws discussed above, there are other limitations that should be taken into account. First, the quasi-experimental design could have introduced bias in the process of selection of schools and their involvement in the training and research. Second, some of the self-reported instruments were not validated against Spanish population and, although back-translation process was employed, the questionnaire may not yield to the different cultural expressions (Berg et al., 2020). Finally, working on social and emotional competencies may have interfered or added an extra load of work to the daily routine of teachers, affecting, thus, their teaching labor and indirectly the students’ monitoring on the acquisition of competencies.

## Conclusion

Even though the study has failed to achieve the expected results in all the studied variables, further research on the advantages of the project Learning to Be in the long term is necessary. Despite this kind of programs can mean an opportunity for the achievement of a school climate with a positive impact, playing an important role in the development of SEL competencies in students (Cohen et al., 2009; Divecha and Brackett, 2020; Mira-Galvañ and Gilar-Corbi, 2020), including a formative assessment in SEL programs might need further development.

## Data Availability Statement

The raw data supporting the conclusions of this article will be made available by the authors, without undue reservation.

## Ethics Statement

This study was reviewed and approved by Ethical Board of the University of Helsinki and Ethical Board of the Universidad Loyola Andalucía. Written informed consent to participate in this study was provided by the participants’ legal guardian/next of kin.

## Author Contributions

OJ prepared the data sets and was in charge of the data analysis. DMR and DR-A described the theoretical framework and were in charge of the literature research. DMR, DR-A, and EM participated in the creation of the Toolkit and the data collection. OJ participated in the training for the L2B intervention group. All authors participated in this manuscript.

## Conflict of Interest

The authors declare that the research was conducted in the absence of any commercial or financial relationships that could be construed as a potential conflict of interest.

## Supplementary Material

Supplementary Table 1 The TIDieR checklist.
